# The evolutionary genetics of highly divergent alleles of the mimicry locus in *Papilio dardanus*

**DOI:** 10.1186/1471-2148-14-140

**Published:** 2014-08-31

**Authors:** Martin J Thompson, Martijn JTN Timmermans, Chris D Jiggins, Alfried P Vogler

**Affiliations:** Department of Life Sciences, Natural History Museum, London, SW7 5BD UK; Department of Zoology, University of Cambridge, Downing Street, Cambridge, CB2 3EJ UK; Department of Life Sciences, Imperial College London, South Kensington Campus, London, SW7 2AZ United Kingdom

**Keywords:** Mimicry, Balanced polymorphism, Supergene, *Engrailed*, Phylogenetics, Molecular evolution

## Abstract

**Background:**

The phylogenetic history of genes underlying phenotypic diversity can offer insight into the evolutionary origin of adaptive traits. This is especially true where single genes have large phenotypic effects, for example in determining polymorphic mimicry in butterflies. Here, we characterise the evolutionary history of two candidate genes for the mimicry switch in the polymorphic Batesian mimic *Papilio dardanus* coding for the transcription factors *engrailed* and *invected.*

**Results:**

We show that phased haplotypes associated with the dominant morphs f. *poultoni* and f. *planemoides* are phylogenetically highly divergent, in particular at non-synonymous sites. Some non-synonymous changes are shared between the divergent alleles suggesting either convergence or a shared ancestry. Gene trees for *invected* do not show this pattern. Despite their great divergence, all *engrailed* alleles of *P. dardanus* were monophyletic with respect to alleles of closely related species. Phylogenetic analyses therefore reveal no evidence for introgression from other species. A McDonald-Kreitman test conducted on a population sample from South Africa confirms a significant excess of intraspecific non-synonymous diversity in *P. dardanus engrailed*, suggesting long-term balanced polymorphism at this locus.

**Conclusions:**

The divergence between *engrailed* haplotypes suggests an evolutionary history distorted by selection with multiple changes reflecting recurrent selective sweeps. The high level of intraspecific polymorphism observed is characteristic of balancing selection on this locus, as expected if the gene *engrailed* is under phenotypic selection for the maintenance of multiple mimetic morphs. Non-synonymous changes in key functional portions of a major transcription factor are likely to be deleterious but if maintained in a dominant allele at low frequency, heterozygosity would reduce the associated genetic load.

**Electronic supplementary material:**

The online version of this article (doi:10.1186/1471-2148-14-140) contains supplementary material, which is available to authorized users.

## Background

A key aim of evolutionary biology is to understand the processes that give rise to novel traits. What is the nature of the genetic changes underlying adaptation? How are new alleles introduced into a population, and how are they maintained in the face of varying types of selection? One way of solving these questions is to study polymorphic species – the genetic control of a polymorphism within species can be used to test hypotheses regarding modification of developmental processes at broader taxic scales [1]. To find the genes responsible, we can study nucleotide variation and search for characteristic signatures that selection will leave in the diversity of alleles in a species [2]. Such ‘signatures of selection’ can corroborate the role of the genes in determining the polymorphism. The identity of these genes will contribute to our understanding of the processes generating and maintaining evolutionary diversity.

Another key question underlying studies of phenotypic evolution is how complex phenotypes come under precise genetic control. Locally polymorphic species pose a particular challenge: multiple phenotypic optima are occupied by individuals from a single population requiring precise determination of the various discrete phenotypes despite mating between individuals of different morphs. Such situations can lead to the evolution of supergenes [3] where a single genetic locus comes to determine the polymorphism. Whatever the exact genomic architecture of the supergene, it seems likely that switching between alternate phenotypes is accomplished by differential regulation of effector genes. The ‘cis-regulatory’ hypothesis for morphological adaptation states that the regulatory regions of genes can evolve at a higher rate (and with fewer constraints) than the protein-coding regions of genes [4, 5]. This hypothesis predicts that morphological evolution will tend to arise through regulatory changes, often in cis-regulatory control of conserved genes involved in developmental processes [6].

Lepidopteran wing patterns are some of the best known examples of adaptive colouration and include some textbook examples of natural selection in action, including industrial melanism in *Biston betularia* and Müllerian mimicry in *Heliconius* butterflies [7–9]. These systems have revealed that wing pattern diversification is controlled by a small number of genes with alleles of large effect [10–12]. When the genomic regions containing these genes are analysed phylogenetically, they often display an evolutionary history that is discordant with that of the rest of the genome, producing topologies that group similar phenotypes together irrespective of species boundaries and geographic structure [13, 14]. In some cases these patterns have resulted from adaptive introgression, or collateral evolution by allele sharing [10, 15]. Reconstructing phylogenies of genomic regions that control phenotypic diversity can therefore be a powerful method for verifying their involvement in generating and maintaining phenotypic diversity, and in increasing our understanding of the processes giving rise to novel and adaptive phenotypes (e.g. [16]). Recent advances in unpicking the supergene underlying Batesian mimicry in *Papilio polytes* [12] offers insight into the nature and function of supergenes. In this case, a single coding region, *doublesex*, was found to determine female polymorphism, possibly through differential expression of isoforms of *doublesex*. High levels of synonymous and non-synonymous polymorphism were found in the *doublesex* coding region and alternative alleles were found to be highly divergent.

**Figure 1:**
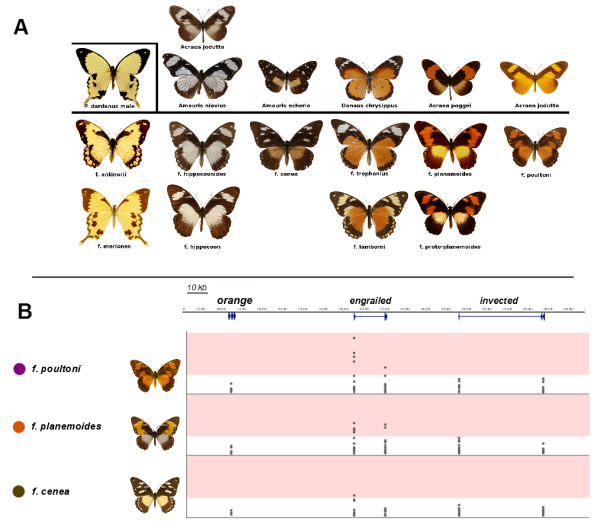
**Phenotypic variation in P. dardanus and the organisation of the H locus.** **(A)** Female forms of *P. dardanus* (below the horizontal line) and examples of their model species in a number of African mimicry rings. The *P. dardanus* male pattern is shown boxed at the left along with male-like female forms. **(B)** Genomic organisation of the *H* locus showing the extent of exons (vertical blue bars) and introns (blue line) of the *en* and *inv* genes along the known sequence of the region. The bottom panels show the significance level for associations of SNP in the respective exons with three dominant morphs. The shading indicates the significance threshold for association. Note that significant association is found only in *en* (mostly exon 1). The figure is reproduced in an altered form from [25].

The African Mocker Swallowtail *Papilio dardanus* is a polymorphic female-limited Batesian mimic. At least 14 different female wing pattern morphs can be distinguished in *P. dardanus* ([17, 18], Figure 1). Wing pattern determination in females of *P. dardanus* maps to a single supergene locus, termed *H* [19]. Dominance of colour morphs is complete for most crosses between sympatric individuals, although there is partial breakdown of dominance when butterflies from different subspecies are crossed [19–23].

The *H* locus has been mapped to a 13.9 cM linkage group and shown to co-segregate with the gene coding for the transcription factor *invected* and possibly its paralogue *engrailed* that maps to the same genomic region [24]. More recent work has used a comparative genomic approach to physically map this candidate region and carry out further recombination and SNP-association analyses to test for differences among morphs in this region [25]. Around 24 genes co-segregate with *H* and are broadly syntenic with previously sequenced lepidopteran genomes. Despite the lack of resolution in positional cloning of *H* using pedigree information, this study furthermore demonstrated that the forms f. *cenea*, f. *poultoni* and f. *planemoides* showed significant association with SNP variants in *engrailed* [25] (see Figure 1). None of the other 23 genes showed these SNP associations with all three phenotypes*,* leading to the conclusion that *engrailed* alone is the prime candidate for the mimicry switch locus, *H*. Additionally, linkage disequilibrium in the region was found to be relatively low, but was high for SNPs within *engrailed*, as would be expected if this locus is a supergene harbouring multiple allelic sites which determine alternative phenotypes and which need to be maintained without recombination to avoid the formation of maladaptive intermediates*.* Finally, the comparative genomics analysis also revealed evidence for balancing selection based on the non-neutral distribution of SNP variation in the *engrailed* coding region [25].

These results indicate that *engrailed* and possibly *invected* are strong candidates for the *P. dardanus* mimicry switch. These genes are highly conserved developmental regulatory genes present in all hexapods [26]. If these genes do indeed determine the wing pattern, nucleotide variation will be affected by selection on the phenotype and therefore may have an evolutionary history discordant with that of unlinked markers, which were shown to follow a predominantly geographic structure [27]. Specifically, phylogenetic analysis of mitochondrial markers in *P. dardanus* has revealed deeply diverging lineages comprising Eastern and Western mainland African clades and an Indian Ocean clade, but no comparable separation has been detected in nuclear markers including the *invected* gene and two linked AFLP loci [27]. We additionally predict that similar phenotypes will share alleles at *H* regardless of their geographic origin; individuals of a given morph are therefore expected to be phylogenetically closely related at loci determining the phenotype (i.e. the *H* locus).

Here we use gene phylogenies for coding regions of *engrailed* and *invected* to study the phylogenetic history of these regions within *P. dardanus* and across the genus *Papilio.* Previous work has also demonstrated that *P. dardanus* forms hybrids in the wild with other *Papilio* species [28] and gene flow from other species may be a significant source of evolutionary novelty [10]. We therefore test the monophyly of *P. dardanus* alleles and the possibility that divergent alleles have undergone introgression, while also establishing a baseline of divergence in these loci beyond the *P. dardanus* clade. A phylogenetic approach will also be useful for the analysis of *engrailed* and *invected* alleles within the *P. dardanus* lineage to characterise the kind of nucleotide divergence distinguishing the various morphs. Given the known LD in the *engrailed* coding region the phase of SNP variation can be computationally inferred to obtain distinct alleles whose variation may be tree like and amenable to phylogenetic inference. Variation at these loci within *P. dardanus* is then further investigated, relative to population divergence in mitochondrial markers. Finally, tests for selection are applied to reveal associations of haplotypes in *engrailed* and *invected* with wing pattern and to give insight into the molecular evolution of this region.

## Materials and methods

**Figure 2:**
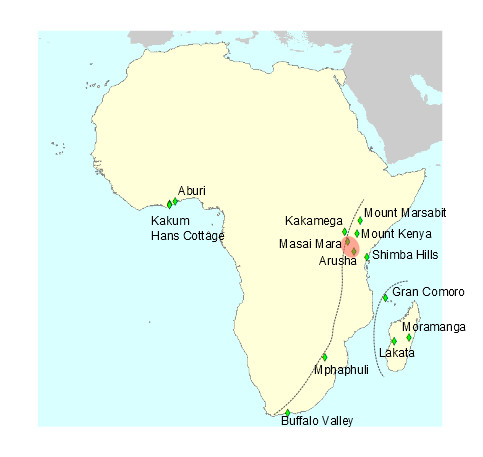
**Sampling locations of** ***P. dardanus*** **used in this study.** Dotted gray lines separate the Eastern, Western and Indian Ocean lineages. The shaded area is *P. dardanus meseres*, believed to be a contact zone between the Eastern and Western mainland lineages.

*Papilio dardanus* specimens were chosen to maximise overlap with previously published taxon sets [25, 27], whilst also adding samples from additional populations from either side of the documented mitochondrial lineage break (East African Rift Valley). Specimens were taken from existing collections of the Natural History Museum (NHM, London), the Afrotropical Butterfly Research Institute Kenya (ABRI, Nairobi) and Stratford Butterfly Farm (Stratford, UK). Additional sampling was carried out in Ghana and South Africa, with further specimens of *P. dardanus dardanus* from Western Kenya (Kakamega Forest) provided by S. C. Collins (ABRI). Sampling locations are plotted in Figure 2. In addition*, P. phorcas, P. constantinus* and other outgroups from the genus *Papilio* were purchased from Stratford Butterfly Farm. A full list of samples used is presented in Additional file 1: Tables S1.

DNA was extracted from legs of fresh or frozen specimens using a Qiagen DNeasy kit. For dried museum collections material (either pinned or stored in envelopes) the protocols of Thomsen et al. [29] were followed. Briefly, this involves removal of a leg of the butterfly, incubation overnight in a buffer with protease and a purification step using a Qiagen QIAquick PCR cleanup kit. Primer sequences [25, 28, 30, 31] are provided in Additional file 2: Table S2. PCR products were bi-directionally Sanger sequenced using ABI technology. Sequences were checked and edited using Geneious (Version 6.1 [32] and aligned using the MAFFT [33] plugin for Geneious with default settings. Numbers of variable sites (including parsimony-informative, synonymous and non-synonymous sites) were calculated for each alignment in SITES [34].

To improve the accuracy of haplotype inference, allelic phase data was directly obtained for 2 individuals per morph through cloning of *engrailed-*derived PCR amplicons with Invitrogen TOPO TA vector and Invitrogen chemically competent *E. coli* grown on kanamycin/LB agar. PCR was performed on picked colonies directly prior to Sanger sequencing (sequences submitted to Genbank, accessions KJ507618-KJ507653). In addition, haplotypic phase data was obtained by sequencing *H-*linked PCR amplicons for 8 specimens (Additional file 3: Table S3) on a Illumina MiSeq (200 bp PE with v2 500 cycle kit, Nextera library prep at an NHM in-house sequencing facility). Resulting reads were trimmed in Geneious version 6.1 [32] using default settings and mapped to a BAC reference (Genbank accession FM995623.2) using Geneious read-mapper ([32], settings: 15% gaps per read, gap size 50, no words >20x, word length 14, index length 12. 30% mismatches per read, maximum ambiguity 4-fold, iterated 5 times). Paired-end information was used to improve the quality of the mapping, using only matches mapping nearby and ignoring multiple matches. The mapping resulted in coverage for each targeted region in excess of 100x, files of reads mapping to the BAC reference have been submitted to SRA (study PRJEB5625, ERP005044). The resulting pileups were used to call haplotypes using GATK read-backed phasing (GATK version 2.3-9, [36, 37]). Only those haplotypes obtained through cloning or Illumina amplicon sequencing were used to represent *P. dardanus* in the genus-level phylogenies (note that this sample includes all studied morphs). Individuals of *P. dardanus* f. *lamborni* were excluded from the present study due to the existence of a duplication of the *engrailed/invected* genome region in this morph [25], potentially confounding phylogenetic or haplotype-level analyses.

### Tree searches

Nucleotide substitution models were obtained from jModeltest (version 2.1, [38, 39], testing 11 substitution schemes, each with 4 categories of rate variation among sites) and using the corrected AIC criterion (AICc) to find the best fitting model. Tree searches were performed under the selected model using PhyML (version 20120412, [39]) with support values assessed using 200 replicates and other settings set to default. Tree statistics (tree length, consistency index, retention index and homoplasy index) were calculated in PAUP* 4.0 [40].

### *Haplotype analysis*within P. dardanus

Sequenced haplotypes from cloned PCR products and Illumina amplicon sequencing were added to *P. dardanus* genotypic data to assist in inferring phase in the remaining specimens using the program PHASE (version 2.1.1, [41, 42]), with settings 400 iterations, thinning interval = 4 and a burn-in of 150. Haplotypes that were not inferred with certainty (P = 1.000) were discarded. A list of samples for which *engrailed* and *invected* haplotypes were successfully inferred is presented in Additional file 4: Table S4. Unrooted phylogenetic trees of *P. dardanus engrailed* and *invected* inferred haplotypes were produced using PhyML as described above. The branch-lengths of these trees were rescaled to reflect the numbers of synonymous and non-synonymous substitutions in HyPhy (version 2.1.2, [43]). Monophyly of the morph-associated *engrailed* alleles was assessed using the Shimodaira-Hasegawa test for tree selection based on constrained trees (monophyly constraint) that keep target alleles as monophyletic against the unconstrained tree using PAUP* 4.0b10 [40]; heuristic search performed under likelihood criterion with 10 random stepwise addition replicates and tree bisection-reconnection branch swapping, using nucleotide substitution model given by jModeltest).

McDonald-Kreitman tests were performed in DnaSP (version 5.10.1, [44]) using 405 bp amplicon *engrailed* haplotypes inferred with PHASE from a population sample of 35 wild-caught specimens of *P. dardanus* subspecies *cenea* (Additional file 5: Table S5). The McDonald-Kreitman test requires a sufficiently divergent outgroup, such that there is little or no shared polymorphism, so we used a specimen of *P. rex*, rather than the more closely related *P. phorcas* or *P. constantinus*.

## Results

### *Phylogeny of*engrailed *and*invected *exons across the genus*Papilio

**Figure 3:**
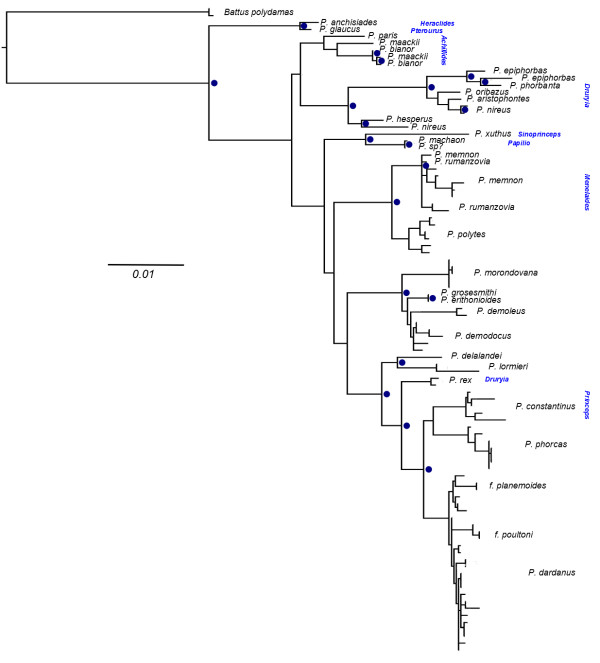
**ML phylogeny of 5’** ***engrailed*** **across the genus** ***Papilio*** **, with a** ***Battus*** **outgroup.** *Papilio dardanus* individuals are represented by haplotypes from either cloning or MiSeq amplicon sequencing. Blue dots indicate nodes with bootstrap support >70%. Bootstraps at intraspecific nodes are ignored.

**Figure 4:**
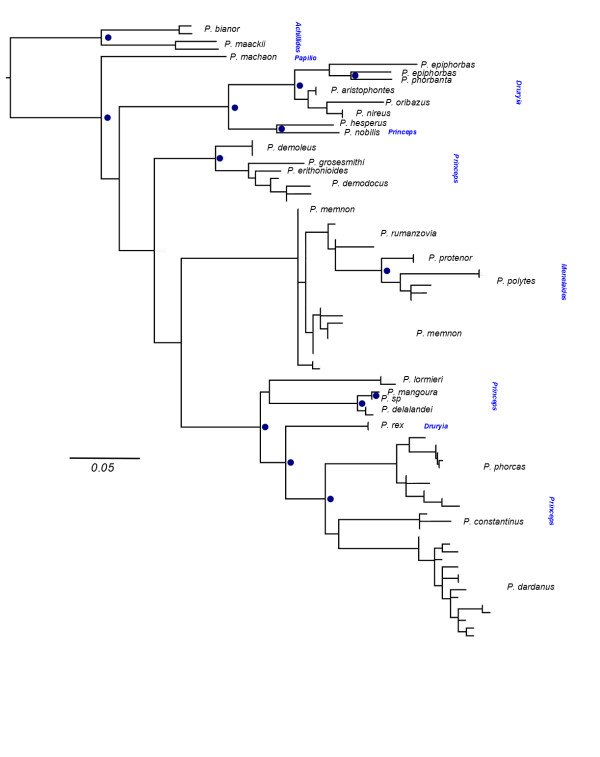
**Maximum likelihood tree with 200 bootstrap replicates for** ***Papilio*** **5’** ***invected*** **.** *Papilio dardanus* individuals are represented by haplotypes from MiSeq amplicon sequencing. Note that *P. demodocus* and *P. memnon* are paraphyletic. Blue dots indicate nodes with bootstrap support >70%. Bootstrap support at intraspecific nodes is not presented.

To study the evolution of the candidate wing-pattern switch genes, we inferred the phylogeny of *engrailed* and *invected* coding sequences across the genus *Papilio* (Figures 3 and 4 respectively). For *engrailed*, we used 93 terminal taxa (30 species) of exon-1 resulting in an alignment of 479 bp of coding region. The analysis places all haplotypes of *P. dardanus* in a clade as sister to its two closest relatives, *P. constantinus* and *P. phorcas*, and reveals a large diversity of alleles within *P. dardanus*. In particular, alleles from individuals of f. *planemoides* and f. *poultoni* represent highly-divergent sequences derived from within a cluster of alleles associated with the other morphs. Monophyly of *P. dardanus* alleles indicates that these alleles have evolved within *P. dardanus*, rather than through introgression from a related species.

Outside of the *P. dardanus* clade, the overall topology is largely consistent with previous findings [45, 46] with the included subgenera, principally *Princeps, Druryia* and *Menelaides* recovered as monophyletic. Within the subgenus *Menelaides,* the species *P. memnon* and *P. rumanzovia* are not reciprocally monophyletic with one specimen of each species in an unresolved position at the base of this clade. Consistent with previous analyses [45] our tree places *P. rex* within subgenus *Princeps*; *P. rex* is therefore corroborated as sister to the clade (*P. dardanus, P. constantinus, P. phorcas*).

The dataset for *invected* consisted of 81 terminals (26 species) within *Papilio* and an alignment of 332 bps. The resulting tree also reveals a monophyletic *P. dardanus* group, with *P. rex* as sister, and is composed of highly divergent alleles that separate the monophyletic *P. dardanus* from its closest relatives *P. phorcas* and *P. constantinus*. The divergences within *P. dardanu*s are more uniform than in the *engrailed* locus and there is no association of haplotypes with particular phenotypes.

### *Intraspecific variation in*P. dardanus engrailed *haplotypes*

**Figure 5:**
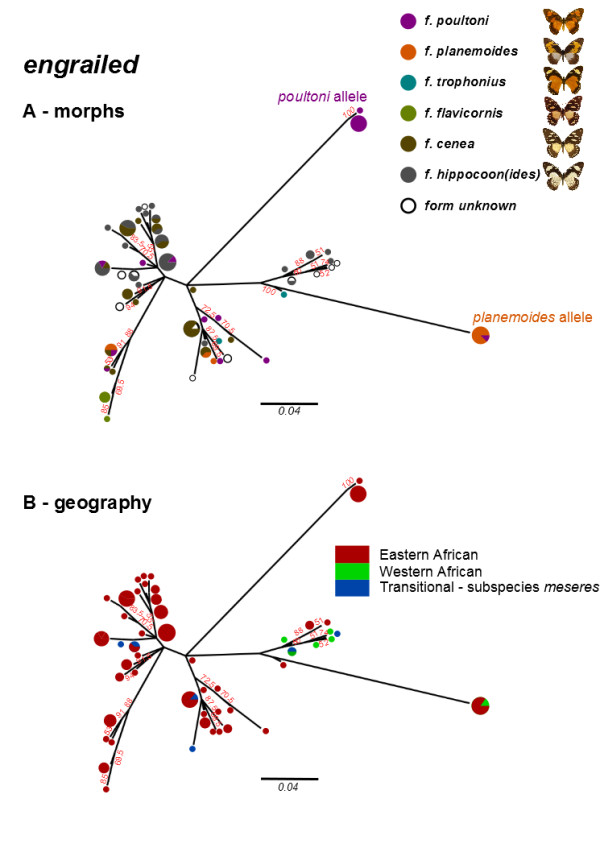
**Maximum likelihood phylogeny of inferred 712 bp haplotypes of** ***engrailed*** **in** ***P. dardanus*** **, labelled by either the morph (A) or the population (B) the haplotypes were sampled from.** Divergent alleles are associated with the dominant forms f. *poultoni* and f. *planemoides*, whilst there is no geographic structure. Percentage bootstrap support values above 50% are labelled in red.

Haplotype inference for *P. dardanus engrailed* resulted in 51 unique haplotypes from 55 individuals inferred successfully for a 712 bp 5` region of *engrailed* from a sample of 174 individuals. The haplotype phylogram (Figure 5) shows highly divergent *engrailed* alleles that were associated with the dominant morphs f. *planemoides* and f. *poultoni*. Out of nine individuals of f. *poultoni* sequenced, eight share two very closely related haplotypes. All six f. *planemoides* sequenced have one allele in common, which is also shared with the single f. *poultoni* specimen lacking the f. *poultoni-*associated allele (BMNH746707). This individual may represent a ‘synthetic *niobe*’, i.e. the heterozygote between the f. *planemoides* and f. *trophonius H* alleles known to produce a phenotype similar to that of f. *poultoni* [20], or it may be f. *salaami*, a form with a similar, but distinguishable phenotype which may be specified by a different allele. Unfortunately the wings of this specimen are too damaged to be certain in its morph assignment. One individual (BMNH746604) of f. *poultoni* was found to be a heterozygote of the two divergent alleles in agreement with the documented dominance of the f. *poultoni* allele over f. *planemoides* [20, 22] and the pattern of SNP association observed for this specimen [25]. All individuals of these morphs possess another allele in addition to the divergent and morph-associated allele, indicating heterozygosity at *H* and consistent with the fact that f. *planemoides* and f. *poultoni* are dominant alleles.

The haplotypes can also be labelled according to the population from which they were sampled (Figure 5B). All of the haplotypes from Western Africa (subspecies *P. d. dardanus*) can be found within a single clade, however this lineage also contains a few Eastern African haplotypes from *P. d. polytrophus*. Subspecies *P. d. meseres* is treated separately as this has been suggested to be a ‘transitional’ or hybrid race where the Eastern and Western lineages meet around Lake Victoria [47–49]. Consistent with this hypothesis, individuals from this region are found to possess haplotypes of both Eastern and Western groups (Figure 5B). Finally, the f. *planemoides-*associated allele is found in individuals from both Eastern and Western African populations, possibly demonstrating a history of this allele independent of the population-level biogeography [27]. In summary, there is some geographic structure within our sampling of *engrailed* alleles, but allelic divergence within *P. dardanus* is deeper than any geographic structure.

### *P. dardanus*invected *haplotypes*

**Figure 6:**
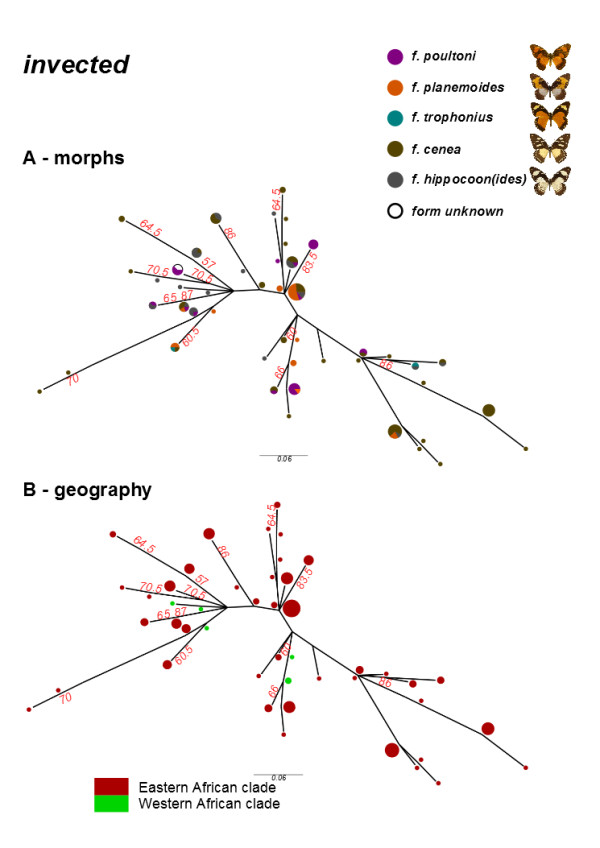
**Phylogeny of inferred haplotypes for** ***P. dardanus invected*** **, labelled by either the morph (A) or the population (B) the haplotypes were sampled from.** Percentage bootstrap support values >50% are labelled in red.

Intra-specific haplotype inference for the 5` region of *invected* (385 bp) was successful for 89 out of 114 individuals, yielding 52 unique haplotypes. The unrooted phylogram (Figure 6) is similar to that for *engrailed* (Figure 5), in that there is variation within the sampled population and there is no geographic structure. However, variation is overall lower than *engrailed* with only 27 variable sites in the nucleotide alignment of 333 positions.

### Nature of changes

**Figure 7:**
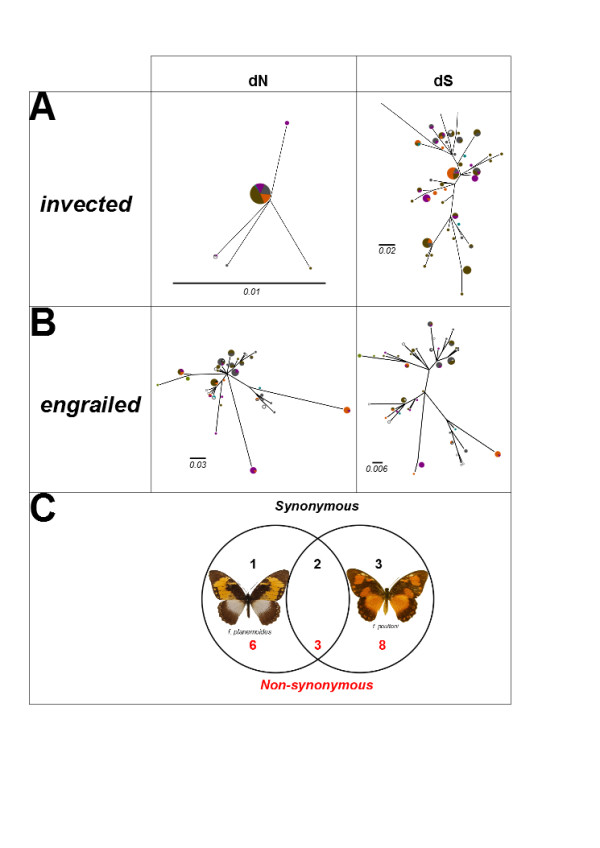
**The structure of genetic variation in invected and engrailed.** Gene trees were established by maximum likelihood for invected **(A)** and engrailed **(B)** haplotypes, separately from non-synonymous and synonymous nucleotide changes. Branch lengths correspond to the number of changes. The area of each pie is scaled in proportion to the frequency of that haplotype among the morphs sampled, and colours of pies reflect proportion of haplotypes recovered from each morph, labelled as in Figure 4. **(C)** Unique SNPs in the two morph-associated alleles relative to the consensus sequence for all other inferred *P. dardanus* alleles.

Haplotype trees for *engrailed* exon 1 with synonymous and nonsynonymous changes mapped demonstrate that much of the divergence of the morph-associated alleles from the rest of the *P. dardanus* alleles is at non-synonymous (replacement) sites (Figure 7). The f. *poultoni* and f. *planemoides* alleles are characterised by multiple unique changes, primarily at replacement sites (Figure 7C). Additionally, the f. *poultoni* and f. *planemoides* alleles share 3 non-synonymous and 2 synonymous changes. To test for a shared origin of the two divergent alleles, we compared the best tree with an alternative topology in which f. *poultoni* and f. *planemoides* were constrained to monophyly. The likelihood of the constrained tree (lnL = -1846.602) was only 0.284 lnL units lower than in the unconstrained tree. This was not a significantly worse (P = 0.98) fit to the data in the SH test, indicating that we cannot rule out a shared origin for these alleles. Unlike the extensive non-synonymous changes in *engrailed*, all changes within the *P. dardanus* complex in *invected* were synonymous.

To test for non-neutral coding evolution within *P. dardanus engrailed,* the McDonald-Kreitman test [50] was applied to inferred *engrailed* haplotypes from a South African *P. d. cenea* population (specimens in Additional file 5: Table S5). We used this population as it was the largest wild-collected sample from a single locality and time point available to us. This population has neither of the dominant morphs f. *poultoni* and f. *planemoides,* but is polymorphic for f. *hippocoonides,* f. *cenea,* f. *natalica* and f. *trophonius.* Haplotypes were inferred for a 405 bp region (the same amplicon targeted for the genus phylogeny), yielding 26 unique haplotypes from 35 individuals. Haplotypes were also inferred for the same region in the outgroup species *P. rex* (2 haplotypes from 1 individual). The test values (Table 1) indicated a significant departure from neutrality, due to a large excess of non-synonymous polymorphisms within *P. dardanus* (P = 0.0128, two-tailed Fisher’s exact test), suggestive of diversifying selection acting on the *engrailed* coding sequence.

**Table 1:** **Segregating and fixed synonymous and non-synonymous differences in the comparison P** ***. dardanus cenea - P. rex***

***P. dardanus cenea – P. rex***	Synonymous	Non-synonymous
**Fixed**	21	4
**Polymorphic**	16	15

## Discussion

We show here that phased haplotypes of *engrailed* associated with wing pattern forms are highly divergent phylogenetically*.* It has recently been shown that sets of SNPs across *engrailed* show strong association with the wing patterns of f. *poultoni* and f. *planemoides* and that this region shows elevated linkage disequilibrium (Figure 1; [25]). The presence of multiple linked SNPs is a prerequisite for the recognition of phylogenetically distinct lineages, and we show here that these morph-associated SNPs define a small number of morph-associated haplotypes. Each individual of these two morphs possesses at least one of these haplotypes, which occupy exceptionally long branches in the haplotype tree, even by comparison to the corresponding sequences across distantly related species of *Papilio* (Figure 3). The divergence of the morph-associated haplotypes is contributed disproportionately by non-synonymous changes. The allele diversity does not follow the strong geographic structure suggested by the mtDNA, which splits the populations in eastern and western branches ([27] and Additional file 6: Figure S1) in roughly the same way as genitalic characters [49], although other nuclear markers also show only weak subdivision ([27] and unpublished). Taken together, these findings strongly suggest that the genomic region around the first exon of *engrailed* diverges from patterns of neutral variation at phylogenetic and genetic levels, which further supports a role of this region for controlling wing pattern variation in *P. dardanus*.

The phylogenetic analysis revealed several details about the evolutionary history of *engrailed* and the adjacent *invected* loci. First, the higher-level analyses of the genus *Papilio* (Figures 3 and 4 respectively) did not yield many surprises; expected subclades were largely recovered for both loci, albeit with generally poor support due to the short (<800 bp) fragment length and high homoplasy (Additional file 7: Table S6). Hence it can be concluded that the *engrailed* and *invected* loci are useful to track the lineage history, and are not generally distorted by selection outside of *P. dardanus*. Another key result is that *P. dardanus* is recovered as monophyletic in the *engrailed* phylogeny, despite the inclusion of the divergent haplotypes, arguing against an origin for divergent alleles in other species. The intraspecific trees of *P. dardanus* alleles were characterised by a largely unresolved polytomy at the base in both loci, with very little internal structure related to geography, confirming the conclusions of [27] based on a more limited sample. Additionally, there is no obvious phylogenetic structure beyond the divergent alleles associated with f. *poultoni* and f. *planemoides* in the sequenced region of *engrailed* (Figure 5). Hence, aside from these divergent alleles, the phylogenies corroborate the findings from the SNP associations [25], that did not relate the recessive morphs to particular SNPs.

The existence of multiple fixed differences in the two divergent alleles is likely a result of recurrent fixation of novel mutations. The two groups of morph-associated alleles may share some changes due to a common ancestry or alternatively, if these non-synonymous changes have a direct functional role, this might represent convergence between the two alleles. Indeed, all of the changes that unite the f. *poultoni* and f. *planemoides* alleles are non-synonymous, consistent with the second hypothesis. However, the observation of an elevated level of non-synonymous intraspecific polymorphism in *P. dardanus engrailed* is not limited to the obvious cases of the morph-associated alleles. The results of the McDonald-Kreitman test provide strong evidence for non-neutral evolution in this gene. This has been observed with the SNP variation in the highly polymorphic Kenyan population used for the initial association studies [25], and is confirmed here for a second population from a different region (South Africa) that does not even include the most divergent alleles. The *engrailed* locus in *P. dardanus* therefore shows a pattern of evolution that differs from the remainder of the genus *Papilio* and also from the adjacent *invected* locus, in that non-synonymous changes have accumulated at a much increased evolutionary rate.

It is highly surprising to find such rapid coding sequence evolution in a gene that is so highly conserved across the arthropods. In particular, one of the changes unique to f. *planemoides* occurs in the conserved EH1 domain of the Engrailed protein, changing the core motif from FSISNIL in all other *P. dardanus* to YSISNIL which confers a change in the otherwise conserved core of FxIxxIL [51]. Given this conserved sequence it seems likely that this change may affect binding of Engrailed to the transcriptional repressor Groucho [52, 53]. Whether or not the coding changes in the two divergent alleles affect the function of the Engrailed protein remains an open question. Similarly, we cannot be certain whether these variant sites are themselves the focus of selection, or whether their fixation is the result of hitchhiking due to linkage with other changes. In the latter case, we hypothesise that favourable selection due to a novel mimetic resemblance outweighs the negative effects of a linked genetic load; selection coefficients for mimicry are likely to be very high [54].

Theory predicts that alleles with a high linked genetic load, as might be conferred by the amino acid changes in the EH1 domain, are likely to be under selection to increase in dominance: linked genetic load is ‘sheltered’ in rare dominant alleles as individuals will nearly always be heterozygous for these alleles [55]. In the Batesian mimicry system of *P. dardanus*, the equilibrium frequency is the abundance of the mimic relative to their model at which fitness is maximised, without predators associating a pattern with palatability rather than toxicity [56]. The equilibrium allele frequency will therefore be on average lower for a dominant allele as compared to alleles further down the dominance hierarchy. Hence, we speculate that whether the coding sequence changes at *engrailed* are in any way functional or not, their likely negative pleiotropic effects may be shielded from selection by the recessive-morph alleles at *engrailed*. This shielding of deleterious mutations through heterozygosity may therefore explain why it is especially the dominant morphs which show such a large excess of non-synonymous mutations.

Finally, we may ask what these results contribute to our understanding of the evolution of the supergene that was hypothesized to underlie the phenotypic variation in *P. dardanus.* The evolution of linkage disequilibrium to maintain co-adapted alleles is a core facet of classical supergene theory [57, 58] and other supergene systems have been shown to possess complex genomic architectures that may act to reduce recombination between supergene alleles [12, 59–61]. Here we show that the genomic signature of selection evident from the phylogenetic trees and increased rates of non-synonymous changes does not extend beyond *engrailed*; we find little evidence for morph-associated variants at linked genes such as *invected*. A similar pattern was seen in the SNP analysis [25], along with the low level of linkage disequilibrium observed elsewhere in the *H* region. The high level of diversity uncovered in a population sample of *P. dardanus* is indicative of negative frequency-dependent selection, as would be expected for a locus underlying polymorphic Batesian mimicry, as selection against over-abundant phenotypes results in a balanced polymorphism, which can be detected by a signature of increased diversity. The existence of multiple fixed changes in these alleles could be the result of recurrent selective sweeps fixing the variants, with either the coding region sites under selection themselves or hitchhiked to fixation as the result of selection on linked variants. The changes might also reflect the build-up of genetic load due to a reduction of recombination as predicted by classic supergene theories of polymorphic mimicry. The previous observation of low linkage disequilibrium beyond the *engrailed* locus [25] means that this predicted effect only affects a single gene which, however, spans a large genomic region of ~70 kb [25].

The findings of high levels of non-synonymous diversity, potentially affecting protein structure, are similar to patterns observed in the *doublesex* coding region in *P. polytes* [12]. Both the *P. dardanus engrailed* locus and *P. polytes doublesex* have alternative alleles associated with different female mimetic morphs and these alternative alleles differentiated by multiple changes with a high ratio of non-synonymous to synonymous SNPs in the coding regions. These two mimetic butterflies have evolved similar systems of Batesian mimetic polymorphism through a complex sequence of changes in single developmentally important genes, although the genes involved are very different.

## Conclusions

The gene *engrailed* is a well-supported candidate for the mimicry switch locus in *P. dardanus.* Following the comparative genomics work of [25], we here investigated the evolutionary genetics of the *engrailed* and adjacent *invected* genes using phased alleles within the *P. dardanus* lineage and also provided the wider geographic and phylogenetic context by including additional populations of *P. dardanus* and other members of the genus *Papilio*. We also expanded the tests for non-neutral variation to a geographically distinct population, along with the use of a less divergent outgroup. These analyses revealed that dominant morphs are associated with highly divergent haplotypes of this gene in *P. dardanus*, with a large proportion of the divergence occurring at non-synonymous sites, but not in other species. In addition, there is no evidence for introgression from other species to explain the high level of divergence from other *P. dardanus* haplotypes. Furthermore, the high levels of non-synonymous polymorphism observed in *P. dardanus engrailed* are consistent with long-term balancing selection, mirroring similar findings in *P. polytes* [12] *.* The study provides new insights into the fascinating evolution of mimetic polymorphisms in the genus *Papilio* and shows that, while the polymorphism is generated by different genomic regions, the evolutionary processes that build up the phenotypic diversity at the genome level are similar between species of *Papilio*.

## Availability of supporting data

DNA sequences are in Genbank under accession numbers given in a supplemental table.

## Electronic supplementary material

Additional file 1: Table S1: Table of samples of *Papilio* used for phylogenetic analysis of *engrailed* and *invected.* (XLSX 41 KB)

Additional file 2: Table S2: Table of PCR primers. (XLSX 8 KB)

Additional file 3: Table S3: Table of samples used for Illumina MiSeq amplicon sequencing to infer haplotypes. (XLSX 9 KB)

Additional file 4: Table S4: Table of samples of *P. dardanus* used for phylogenetic and haplotype-level analysis. (XLSX 13 KB)

Additional file 5: Table S5: Table of samples of *P. dardanus cenea* from Mpaphuli Cycad Reserve population used in population genetic analyses. (XLSX 17 KB)

Additional file 6: Figure S1: Maximum likelihood phylogeny of cytohrome b amplicon within the *P. dardanus* species group. *P. dardanus* is presented as sister to a clade of *P. phorcas* and *P. constantinus*, consistent with the engrailed phylogeny in Figure 3. This phylogeny recovers 3 deeply-coalescing lineages within P. dardanus, consistent with the mitochondrial phylogenies of Clark and Vogler [27]. (PNG 106 KB)

Additional file 7: Table S6: Table of summary statistics for alignments, details of nucleotide substitution models used in maximum likelihood tree searches and summary statistics for the resulting phylogenies. (XLSX 9 KB)
