# Supplementary figures and images for: The evolutionary genetics of highly divergent alleles of the mimicry locus in Papilio dardanus
Source: BMC Evol Biol. 2014 Aug 31;14:140. doi: 10.1186/1471-2148-14-140 (PMC4262259; doi:10.1186/1471-2148-14-140)

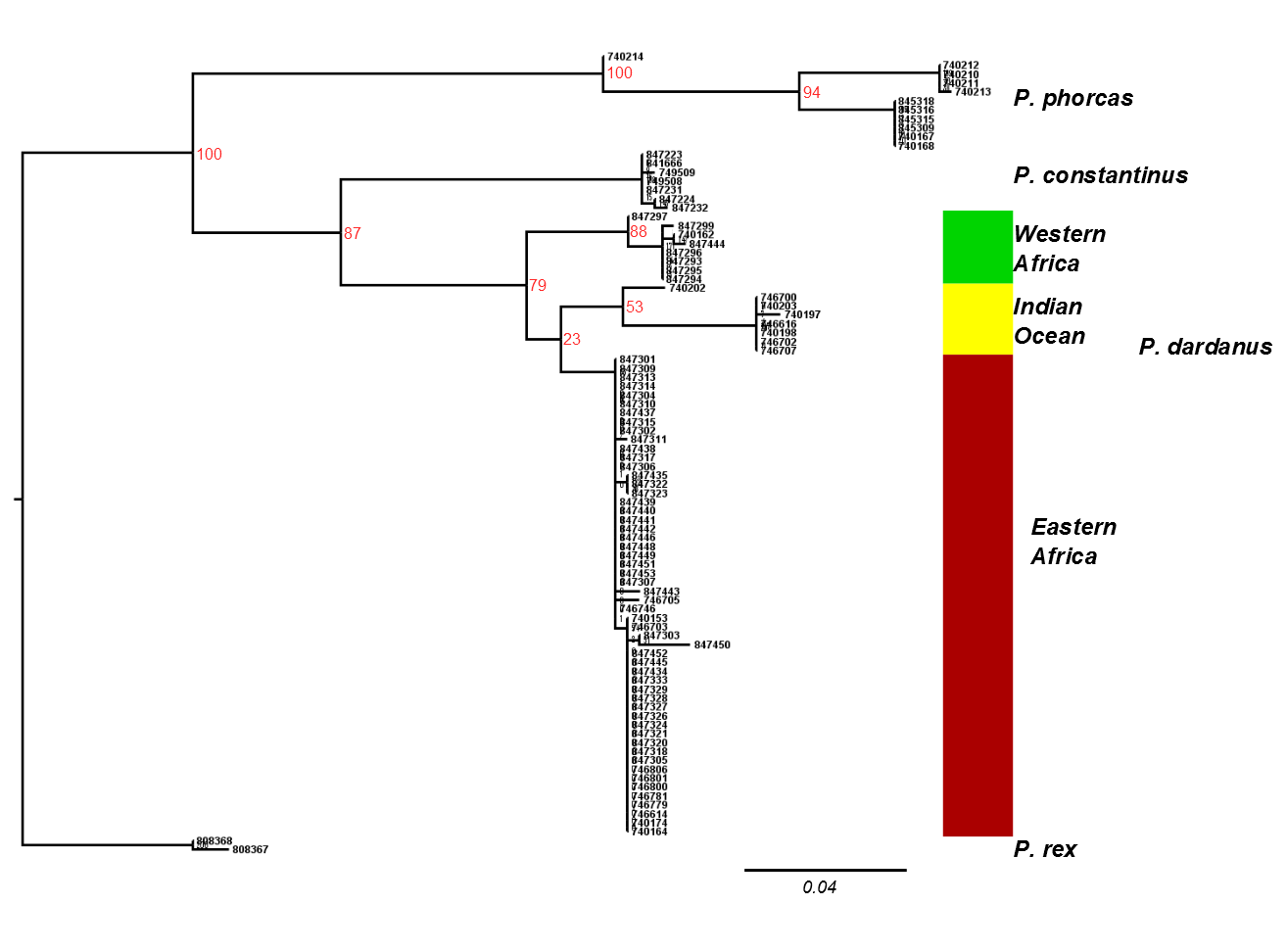

Supplement: Supplementary file 6 — Additional file 6: Figure S1: Maximum likelihood phylogeny of cytohrome b amplicon within the P. dardanus species group. P. dardanus is presented as sister to a clade of P. phorcas and P. constantinus, consistent with the engrailed phylogeny in Figure 3. This phylogeny recovers 3 deeply-coalescing lineages within P. dardanus, consistent with the mitochondrial phylogenies of Clark and Vogler [27]. (PNG 106 KB) [file 12862_2014_2638_MOESM6_ESM.png]
